# *XModeScore*: a novel method for accurate protonation/tautomer-state determination using quantum-mechanically driven macromolecular X-ray crystallographic refinement

**DOI:** 10.1107/S2059798316002837

**Published:** 2016-03-30

**Authors:** Oleg Borbulevych, Roger I. Martin, Ian J. Tickle, Lance M. Westerhoff

**Affiliations:** a QuantumBio Inc., 2790 West College Avenue, State College, PA 16801, USA; b Astex Pharmaceuticals, 436 Science Park, Milton Road, Cambridge CB4 0QA, England

**Keywords:** X-ray crystallography, quantum-mechanics X-ray refinement, ligand strain, high-throughput crystallography, protonation states, tautomers, difference density *Z* score, structure-guided drug discovery, structure-based drug discovery, *XModeScore*

## Abstract

Gaining an understanding of the protein–ligand complex structure along with the proper protonation and explicit solvent effects can be important in obtaining meaningful results in structure-guided drug discovery and structure-based drug discovery. Unfortunately, protonation and tautomerism are difficult to establish with conventional methods because of difficulties in the experimental detection of H atoms owing to the well known limitations of X-ray crystallography. In the present work, it is demonstrated that semiempirical, quantum-mechanics-based macromolecular crystallographic refinement is sensitive to the choice of a protonation-state/tautomer form of ligands and residues, and can therefore be used to explore potential states. A novel scoring method, called *XModeScore*, is described which enumerates the possible protomeric/tautomeric modes, refines each mode against X-ray diffraction data with the semiempirical quantum-mechanics (PM6) Hamiltonian and scores each mode using a combination of energetic strain (or ligand strain) and rigorous statistical analysis of the difference electron-density distribution. It is shown that using *XModeScore* it is possible to consistently distinguish the correct bound protomeric/tautomeric modes based on routine X-ray data, even at lower resolutions of around 3 Å. These X-ray results are compared with the results obtained from much more expensive and laborious neutron diffraction studies for three different examples: tautomerism in the acetazolamide ligand of human carbonic anhydrase II (PDB entries 3hs4 and 4k0s), tautomerism in the 8HX ligand of urate oxidase (PDB entries 4n9s and 4n9m) and the protonation states of the catalytic aspartic acid found within the active site of an aspartic protease (PDB entry 2jjj). In each case, *XModeScore* applied to the X-ray diffraction data is able to determine the correct protonation state as defined by the neutron diffraction data. The impact of QM-based refinement *versus* conventional refinement on *XModeScore* is also discussed.

## Abstract 2

*XModeScore* determines the correct protomeric/tautomeric state or mode of active-site residues along with any bound ligand(s) using quantum-mechanics-based X-ray refinement followed by post-refinement scoring based on a combination of energetic strain (or ligand strain) and rigorous difference electron-density analysis.

## 1. Introduction   

Within structure-guided drug discovery (SGDD) and structure-based drug discovery (SBDD), accurate understanding of the protein–ligand complex structure, including the relevant proper protonation, is significant for obtaining meaningful results from docking/scoring, thermodynamic calculations, active-site exploration, lead optimization and, ultimately, medicinal chemistry (Pospisil *et al.*, 2003 ▸). The most ubiquitous element in the universe is hydrogen, and these protons are critical for exploring the chemistry within the active site. For example, in the drug Mirapex, which is used to treat the symptoms of Parkinson’s disease, the important chemical activity is conferred by a single aminothiazole tautomeric state rather than an alternative imino tautomer (Varga *et al.*, 2009 ▸); thus the selection of the wrong state during drug design would lead to irrelevant findings. This situation is not uncommon, and drug discovery frequently hinges on the determination of one state *versus* another (Martin, 2010 ▸).

The most common method for structure determination in SGDD/SBDD is macromolecular X-ray crystallography. Unfortunately, an intrinsic problem of X-ray crystallography is its inability to explicitly detect H atoms, even at resolutions close to atomic, because the H atom has the weakest scattering power for X-rays among all elements (Rupp, 2009 ▸). H atoms are small, and their electrons are shifted towards the heavy atoms to which they are bound. Hence, it is generally extremely difficult to experimentally determine the protonation or tautomeric state of both the ligand and the surrounding active site. Protonation states can be unambiguously established using neutron diffraction because the neutron scattering length of deuterium is similar to that of heavy atoms (Bacon, 1975 ▸). Thus, the scattering by hydrogen/deuterium is comparable to that by other atoms in macromolecular structures. However, the prime disadvantage of this method that seriously limits its practical application is the considerable weakness of the neutron beam, leading to reliance on very large crystals and long exposure times for the collection of data of sufficient precision. Furthermore, H atoms have a negative scattering length, in contrast to the isotope deuterium (D); thus, the presence of H atoms gives rise to a cancellation during refinement against neutron data, reducing the sensitivity of the method (Afonine *et al.*, 2010 ▸). Hence, complete deuteration of the sample crystal is highly desirable in a neutron diffraction experiment, but is practically difficult to achieve, since exchangeable protons constitute only about one quarter of all H atoms (Shu *et al.*, 2000 ▸). Overall, neutron diffraction is rarely feasible within industrial SBDD settings.

In addition to the experimental limitations of X-ray crystallography with respect to proton detection, the stereochemical restraints used in conventional refinement are generally rudimentary in nature and do not account for interactions such as hydrogen bonds, dispersion, electrostatics, polarization and charge transfer (Adams *et al.*, 2010 ▸; Kleywegt, 2007 ▸; Kleywegt *et al.*, 2003 ▸). The *DivCon* linear scaling, quantum-mechanics (QM), semiempirical quantum-mechanics (SE-QM) and molecular-mechanics (MM) toolkit (Dixon & Merz, 1996 ▸, 1997 ▸; QuantumBio Inc., http://www.quantumbioinc.com) has been shown to capture the interactions between a target and its ligand(s) (Diller *et al.*, 2010 ▸; Raha *et al.*, 2005 ▸, 2007 ▸; van der Vaart & Merz, 1999 ▸; Zhang *et al.*, 2010 ▸). To address the deficiencies in conventional macromolecular refinement, we previously integrated *DivCon* with the *PHENIX* crystallographic package (Adams *et al.*, 2010 ▸) to replace the conventional stereochemical restraint method with a more accurate quantum-based energy functional in ‘real time’ during refinement (Borbulevych *et al.*, 2014 ▸; QuantumBio Inc.). Traditionally, we think of X-ray refinement as a balancing act between two components: the energy functional and the experimental density. However, with a more accurate (*e.g.* more trustworthy) functional, we can consider the input model, and its complement of atoms, as a third component. The success, as measured by agreement between the final model and experimental density, of an X-ray refinement campaign therefore depends on accuracy in all three components, and if upon completion of refinement there is disagreement between the model and the experimental density, this disagreement could be attributed to deficiencies in any of the components. For example, the functional could be missing a key interaction exhibited in the structure, there could be artifacts in the experiment, or the input model could be in an incorrect protonation state, thereby producing incorrect geometry. With *PHENIX*/*DivCon*, we are able to more accurately capture the key interactions within a protein–ligand complex, including hydrogen bonds, dispersion, electrostatics, polarization, charge transfer, metal coordination *etc.* At the same time, crystallographers with greater proficiency and automation power are able to obtain better experimental densities. However, the question remains: can one create an approach or method that allows one to conclusively show which protonation state is most prevalent within the natural, biological structure?

To answer this question, we consider the fact that even though the H atom does not effectively scatter X-rays, with a more accurate functional we can observe the effects of these protons on the surrounding heavy atoms to determine whether or not the input protonation model is correct. By way of analogy, in 1845, John Couch Adams mathematically predicted the existence of the planet Neptune before its direct observation was made based upon unexplained perturbations in the orbit of the neighboring planet Uranus (Sampson, 1904 ▸). With experimental X-ray methods, we cannot directly observe H atoms; however, using the QM/MM functional we are able to observe the influence of H atoms on the heavy atoms (carbon, nitrogen, oxygen) to which they are bound. Movements of such heavy atoms during QM/MM refinement results in certain changes in the electron-density maps, such as increasing/decreasing difference density peaks and correlation coefficients. The protomer/tautomer that produces the best fit to the experimental data can then be chosen based on statistical treatment (Tickle, 2012 ▸) of the results of the refinement of the structures with all possible protonation states.

With this hypothesis in mind, we developed the *XModeScore* technique, which couples the more advanced QM/MM methods in our refinement tool with a statistical comparison of the final model *versus* experimental density in order to determine whether or not the model is reflective of the actual chemistry within the structure. As opposed to score functions used in other fields, such as the affinity prediction functions used in docking/scoring algorithms, *XModeScore* ‘scores’ the various protonation modes using X-ray density.

## 2. Methods   

### 2.1. Validation method and structure selection, preparation and refinement   

Neutron diffraction does not suffer from the same deficiencies as X-ray diffraction with regard to proton scattering, suggesting that these models can serve as ‘gold standards’ with which X-ray results can be compared. Further, since the *XModeScore* method is directly dependent upon the crystal, the method is sensitive to the actual protonation state found within that crystal, and therefore experimental conditions are important considerations. With this in mind, in order to choose a validation set we focused on those structures which (i) have both an X-ray diffraction model and a neutron diffraction model, (ii) have crystallization conditions (*e.g.* pH, solvent, temperature* etc.*) which are approximately identical between the X-ray experiment and the neutron experiment, (iii) are complexed with chemically relevant or pharmaceutically interesting ligands and (iv) include deposited structure factors. The neutron diffraction model also had to be of a high enough quality that the key protonation states could be determined. Therefore, from the 88 neutron diffraction structures available in the PDB at the time of writing, the following three models were chosen.

(i) The AZM–human carbonic anhydrase II (HCA II) complex: neutron, PDB entry 4g0c; X-ray, PDB entry 3hs4 (Fisher *et al.*, 2012 ▸).
(ii) The 8HX–urate oxidase complex: neutron, PDB entry 4n9m; X-ray, PDB entry 4n9s (Oksanen *et al.*, 2014 ▸).
(iii) The PD-135,040–aspartic proteinase complex: neutron, PDB entry 2vs2; X-ray, PDB entry 2jjj (Coates *et al.*, 2008 ▸).

A second HCA II X-ray model, PDB entry 4k0s (A. Biswas, D. West, M. Pinard & R. McKenna, unpublished work), was also selected in order to demonstrate the impact of resolution on *XModeScore* results.

The X-ray structures, along with their structure factors, were downloaded from the Protein Data Bank (PDB). H atoms were added to protein residues, water molecules and ligands using *Protonate*3*D* (Labute, 2009 ▸) as implemented in *MOE*2013 from Chemical Computing Group. Likely protomer/tautomer states were automatically generated with *MOE*2013 using the *WashMoleculeMOE* Scientific Vector Language (SVL) function. Since *Protonate*3*D* settles on a single protomeric/tautomeric state, after execution of *WashMoleculeMOE* each candidate protomeric/tautomeric state was fixed and *Protonate*3*D* was re-executed on the structure in order to ‘propagate’ proton addition/subtraction (along with corresponding residue rotameric flips) based upon each tautomer or protomer. In this way, protons are added/changed within the active site to match or counterbalance hydrogen-bond changes within the ligand. QM region refinement as detailed previously (Borbulevych *et al.*, 2011 ▸, 2014 ▸) was conducted on each structure using libQB (*DivCon* build-2577) incorporated into the *PHENIX* package v.1.9-1692 (Adams *et al.*, 2010 ▸). The PM6 semiempirical QM Hamiltonian (Stewart, 2009 ▸; Řezáč *et al.*, 2009 ▸) was used for each QM region, where each QM region was centered around the AZM ligand in PDB entries 3hs4 and 4k0s, the 8HX ligand in PDB entry 4n9s and the key catalytic residue Asp215 in PDB entry 2jjj. For the region refinement, the default radii of 3.0 and 2.5 Å for the main and buffer regions, respectively, were used. To explore the impact of resolution on each refinement and score, each structure was refined at several levels of data-set truncation using the *phenix.refine* keyword ‘xray_data.high_resolution=X’, where X refers to the desired high-resolution cutoff in Å.

In addition to QM-based X-ray refinement, conventional (*i.e.* non-QM) refinements using the same version of *PHENIX* were also performed for each case in order to explore the impact of the refinement method on the *XModeScore* results. The necessary CIF files for each protonation/tautomer state were generated using both the *electronic Ligand Builder and Optimization Workbench* (*eLBOW*) module (Moriarty *et al.*, 2009 ▸), which generates restraints using quantum mechanics, and the *Grade* web server (http://grade.globalphasing.org), which produces *Mogul* CIFs based on coordinate data from the Cambridge Structural Database (CSD). The metal restraints around Zn were incorporated in the conventional refinements of the AZM structure as generated with the *PHENIX* program *ReadySet!*.

### 2.2. *XModeScore*: scoring procedure   

The overall goal of *XModeScore* is to determine which protonation or tautomer form is found in the experimental structure. After refinement, each structure was scored based on a combination of metrics which take into account both the structural characteristics of the ligand and its fit within the active site, as well as quality indicators of its agreement with the crystallographic electron density. The local ligand strain energy (SE) serves as an important quality indicator of protein–ligand structures because it shows how much strain the ligand must accept to bind to the protein. The SE or *E*_Strain_ is defined as the difference between the energy of the isolated ligand conformation and the protein-bound ligand conformation and is computed (Fu *et al.*, 2012 ▸) according to
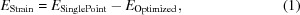
where *E*_SinglePoint_ is the single-point energy computed for the ligand X-ray geometry and *E*_Optimized_ is the energy of the optimized ligand that corresponds to the local minimum. The strain energies in this work have been calculated using the PM6 Hamiltonian (Řezáč *et al.*, 2009 ▸; Stewart, 2009 ▸) as implemented in *DivCon*.

The experimental quality indicator component of *XModeScore* is a measure of the model accuracy or how well the model would have predicted the data. The generally accepted quality metric of the X-ray electron (or neutron) density is the real-space correlation coefficient (RSCC; Rupp, 2009 ▸). The RSCC reflects the degree of correspondence between the experimental (observed) and calculated electron densities. However, as argued previously (Tickle, 2012 ▸), the RSCC correlates both with the accuracy and with the precision of the structure model, and it is not possible to say to what extent the RSCC reflects the accuracy of a given model owing to the variable contribution from the precision component.

On the other hand, the real-space difference density *Z* score for a point difference density value (Tickle, 2012 ▸) defined in (2) provides a more sophisticated quality indicator since it measures the accuracy of the model,
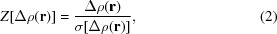
where Δρ(**r**) is the difference density at the coordinate vector **r** expressed as the real Fourier transform (Read, 1986 ▸), 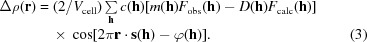
Here, the sum is over observed reflections with index vector **h** symmetry-expanded to a complete hemisphere in reciprocal space, *F*_obs_(**h**) and *F*_calc_(**h**) are the observed and calculated structure-factor amplitudes, respectively, φ(**h**) is the phase calculated from the model, *c*(**h**) is the centricity factor (1 for centric reflections or 2 for acentric reflections), *m*(**h**) is the expected cosine of the phase error, *D*(**h**) is a correction factor for errors in the model and **s**(**h**) is the scattering vector.

In (2), σ[Δρ(**r**)] is the standard deviation of the difference density, which is the standard measure of random error and is therefore a pure precision metric. The *Z* score of the difference density is a measure of the residual nonrandom error and thus is a pure accuracy metric. However, a single minimum or maximum value of the difference density might not be statistically sound, as it is easy to overinterpret the significance of such a *Z* score (Tickle, 2012 ▸). Difference density *Z* values should approach a normal distribution of random errors with zero mean and unit standard deviation as the quality of the model improves, and the presence of significant positive or negative peak outliers that deviate from the expected distribution indicate problems with the model. Therefore, rather than using the point density at the atom center, or a single minimum or maximum value for each atom taken over all grid points covering the atom, it is more reliable to compute the standard chi-square (χ^2^) statistic for a subset of the absolute negative values, and similarly for the positive values, of the density at the grid points covering an atom, assuming independent and identically distributed (iid) random variables. In each case the selected subset starts at the *k*th value in increasing order of magnitude,
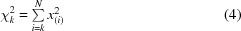
where *x*_(*i*)_ is the *i*th normal order statistic (*i.e.* postulating the null hypothesis of a normal distribution) of the |*Z*(Δρ)| scores for the negative and positive values, respectively [*i.e.* in each case the *i*th value after sorting each array of |*Z*(Δρ)| values in increasing order of magnitude].

Thus, all such grid-point density values become potentially relevant during the evaluation of the ZDD metric, which is a measure of the difference density of an atom that takes into account its variation over the entire atomic volume (6*a* [Disp-formula fd6] and 6*b* [Disp-formula fd7]). Clearly, we do not know *a priori* which of the density values are significant: if we choose too few we may lose information, but if we choose too many and add noise then χ^2^ will lose significance. Therefore, it is reasonable to sum only the subset of values of *x* ^2^_(*i*)_ that maximizes the probability *p*_max_ over *k*,
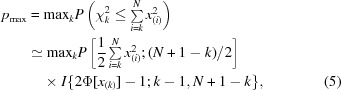
where the function *P* is the lower normalized gamma function representing the cumulative distribution function (CDF) of χ^2^ *_k_*. In practice, the CDF is computed as the complement (1 − *P*) to avoid problems with numerical precision for values of the function *P* near unity, *i.e.* the most relevant values for the present purposes. The second function, *I*, which is also computed as the complement in practice, is the normalized incomplete beta function (CDF of a normal order statistic; Gibbons & Chakraborti, 2010 ▸) which accounts for the ‘multiple comparisons’ correction (Yuriev & Ramsland, 2013 ▸). It is worth remarking that in the special case of *k* = *N*, where the maximal probability *p*_max_ occurs when only the single maximum absolute density value is used, the function *I* becomes the Dunn–Šidák correction (Šidák, 1967 ▸). Another special case occurs for *k* = 1, where the maximal probability occurs when all density values are used. In this case there is no ‘multiple comparisons’ correction, so the function *I* is then exactly 1 and the combined function reduces to the CDF of χ^2^ for *N* degrees of freedom, as expected. In this way, the probability *p*_max_ makes no assumptions about the spatial distribution of significant grid-point values in the vicinity of an atom (*e.g.* whether there is a single sharp maximum, a broad maximum or multiple maxima). Rather, the value of *p*_max_ adapts to the actual distribution and attempts to quantify the probability that the distribution of grid-point values could not have arisen purely from random variations.

ZDD is evaluated as the two-tailed normal *Z* score corresponding to the maximal value *p*_max_ over *k* of the cumulative probability of χ^2^ *_k_* derived from (4) and (5), 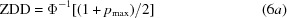
or
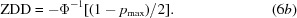
Here, the function Φ is the CDF of the normal distribution [so 2Φ(*|Z*|) − 1 is the CDF of the half-normal distribution of the absolute value of a normal variate *Z*] and Φ^−1^ is the inverse function (*i.e.* the value of *Z* corresponding to a given probability). The form (6*b*) is preferred because the complement (1 − *p*_max_) of the probability was calculated in the previous step.

ZDD also depends on the radius *r*_max_ enclosing the atomic density grid points; this is determined from the radius integral
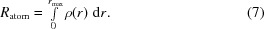
The radius *r*_max_ corresponds to the value of the radius integral *R*_atom_ that is 95% of the theoretical value at infinite radius (Tickle, 2012 ▸). For this purpose, the calculated atomic density function *ρ*(*r*) is determined from the spherically averaged real Fourier transform according to
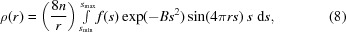
where *n* is the fractional occupancy of the atom, *s*_min_ = 0.5/*d*_max_ depends on the maximum *d*-spacing (or low-resolution limit) *d*_max_ and *s*_min_ = 0.5/*d*_max_ similarly on the minimum *d*-spacing (or high-resolution limit) *d*_min_, *f*(*s*) is the atomic scattering factor for X-rays as a function of *s* and *B* is the isotropic displacement parameter (*B* factor). Thus, the width of the atomic density function (and hence *r*_max_) will be greater at lower resolution and for larger values of the *B* factor, in line with what one expects to see in an electron (or neutron) density map. Where the densities of adjacent (*i.e.* bonded) atoms overlap, the densities at the grid points in the electron-density map are partitioned in proportion to the atomic densities calculated from (8).

To avoid oversampling the density values at the grid points, which would invalidate the iid assumption made above, the set of density values are resampled according to the Shannon–Nyquist theorem (Shannon, 1949 ▸). This theorem states that the density values at the grid points are statistically independent when the sampling interval is at least *d*_min_/2, where *d*_min_ is the minimum *d*-spacing of the data used in the computation of the map. Typically, maps are sampled at an interval of not more than *d*_min_/4 for accurate interpolation and to avoid missing important features, so the map would need to be resampled for the statistical calculations at about every second grid point in each direction. However, since resampling the map in three dimensions might lose information such as significant outliers, the density values are sorted by increasing value as a one-dimensional array and then resampled, keeping only a fraction (*e.g.* 1/8).

The set of negative density values then yields a metric that we call ‘ZDD−’ and the set of the positive densities yields the metric ‘ZDD+’. Therefore, the effects of negative difference density, owing to incorrectly positioned atoms, and positive difference density (perhaps owing to an incorrectly typed atom) can be separately identified. The ZDD− and ZDD+ metrics are also taken together to give a final combined ZDD metric defined as
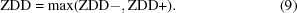
The lowest ZDD in the series of ligand tautomeric forms allows us to choose the best form or protonation state that demonstrates the closest match with experimental density. Then, with both QM-SE and ZDD in hand, the overall score of the tautomer form *i* can be calculated according to 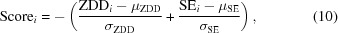
where μ is the mean value and σ is the standard deviation of the corresponding array of data (ZDD or SE). For example, the SE array contains SE values for all tautomers included in the calculations. The highest Score_*i*_ corresponds to the best tautomeric form *i* that fits both the SE and the ZDD criteria.

## 3. Results   

### 3.1. The protonation state of AZM bound to human carbonic anhydrase II: PDB entry 3hs4 at 1.1 Å resolution   

**Figure 1:**
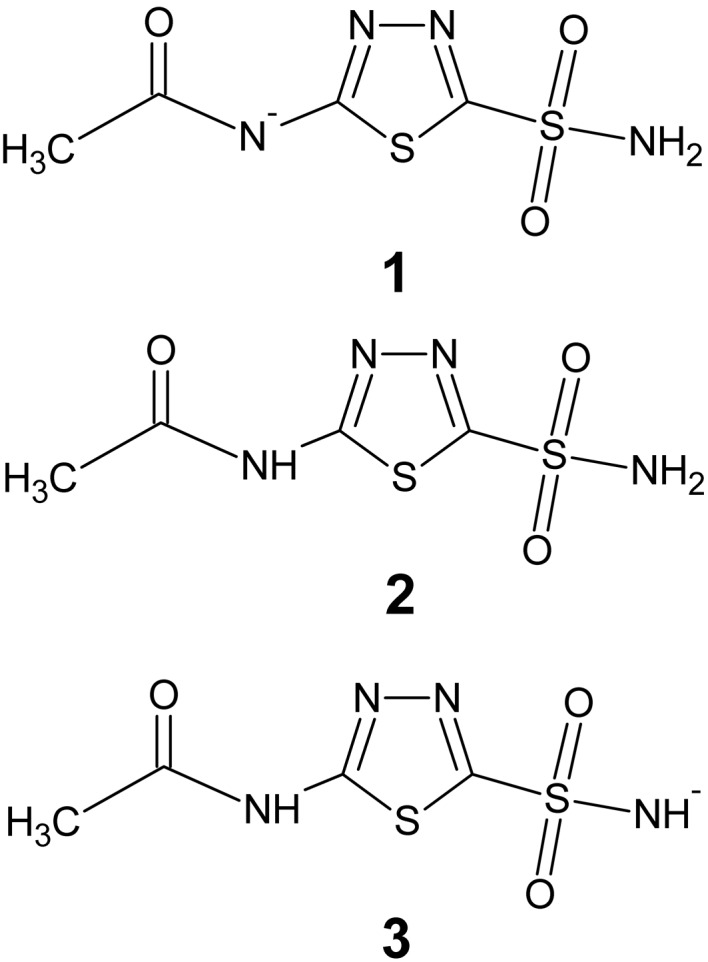
Possible binding modes of the drug AZM.

Human carbonic anhydrase II (HCA II), which catalyzes the hydration/dehydration of carbonates, is involved in numerous metabolic processes including CO_2_ transport and pH regulation and is therefore considered to be an important target for drug design (Krishnamurthy *et al.*, 2008 ▸; Merz & Banci, 1997 ▸). The drug acetazolamide (AZM), sold under the name Diamox, is a high-affinity inhibitor of HCA II that is used to efficiently treat a number of medical conditions (United States Pharmacopeia, 1995 ▸; Moldow *et al.*, 1999 ▸) such as altitude sickness, hypertension and glaucoma. It binds to the Zn atom of the enzyme *via* the N atom of the sulfonamido group. Zn is located in the catalytic center of HCA II and adopts a tetrahedral coordination, making coordination bonds to N atoms of His94, His96 and His119. AZM can exist in two protonation states and several tautomeric forms, which are depicted in Fig. 1 ▸. However, even high-resolution X-ray diffraction studies have failed to determine which form of AZM is actually involved in the enzyme interaction (Sippel *et al.*, 2009 ▸; Fisher *et al.*, 2012 ▸). Conventional protonation-determination methods, such as analysis of the bond-length distribution (Ahmed *et al.*, 2007 ▸), also failed in the case of AZM (Fisher *et al.*, 2012 ▸). It was only with a recent neutron diffraction study (Fisher *et al.*, 2012 ▸) that it was established that AZM exists in form **3**, which includes the negatively charged sulfonamido (SO_2_NH) group bound to zinc.

**Figure 2:**
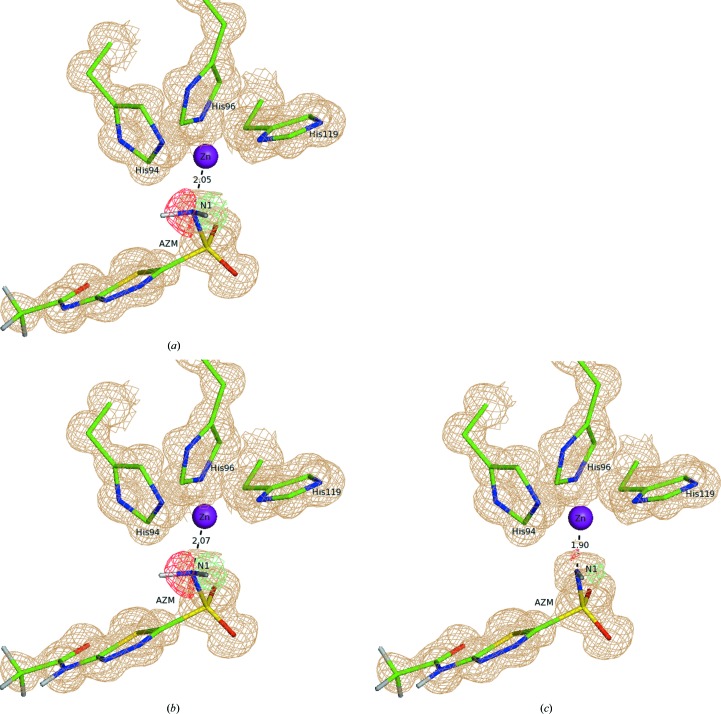
The coordination sphere of Zn in the catalytic center of HCA II with a bound AZM molecule in three alternative binding modes **1** (*a*), **2** (*b*) and **3** (*c*) after QM refinement of the PDB structure 3hs4. The difference density around the key N1 atom of the sulfonamido group of AZM is contoured at the 3.5σ level.

We challenged *XModeScore* with three structures which include three possible forms of AZM beginning with PDB entry 3hs4 (Sippel *et al.*, 2009 ▸; Table 1 ▸ and Fig. 2 ▸). The results indicate that form **3**, the correct protonation state according to the neutron diffraction experiment, is indeed the superior form, dominating in both components of scoring. There is a significant difference in the score for form **3** (2.72) and the score for the second-best option (−0.74), which corresponds to form **2**. It is important to note that the ZDD of form **3** is almost half that of the other two forms, which suggests that structure of tautomer **3**, with the negative charge on the N1 atom bound to zinc, is more consistent with the experimental structure amplitudes than are the forms with an amino group at this position. The difference density maps obtained after QM refinement show that the negatively charged N1 plays a crucial role in binding to HCA II. In particular, large negative/positive peaks of the difference density are seen around the N1 atom for the tautomers **1** and **2** and effectively explain the larger magnitude of ZDD observed for the former tautomer states compared with form **3**. Furthermore, analysis of the bond-length distribution around zinc after QM refinement (Table 2 ▸) shows that the Zn—AZM N1 bond in tautomer **3** (1.90 Å) is much shorter than the length of this coordination bond in the other two tautomers (2.05 and 2.07 Å) and is also shorter than the average length of 2.00 (2) Å for the Zn—N bond type (Harding, 1999 ▸). Nevertheless, such a binding geometry of the ligand AZM with a shortened Zn—N1 bond seen in form **3** agrees much better with the experimental data. Specifically, the atomic ZDD for the N1 atom in form **3** is fourfold lower than the corresponding values observed for the tautomers with a Zn—NH_2_ bond.

### 3.2. Effect of resolution truncation on the predictability of the tautomer by *XModeScore*   

Generally, at lower resolution less detail about the crystal model is revealed from experimental data and these experimental data are less sensitive to the model nuances. Hence, the resolution of data sets may affect not only the absolute values of ZDD but also the difference in tautomer scores. The latter is crucial as it determines the ability to distinguish the top tautomeric form from the rest of the candidates in *XModeScore*. To determine how the resolution affects the predictability of *XModeScore*, we carried out stepwise truncation of the original data set 3hs4 followed by repetition of the scoring protocol at each resolution level.

**Figure 3:**
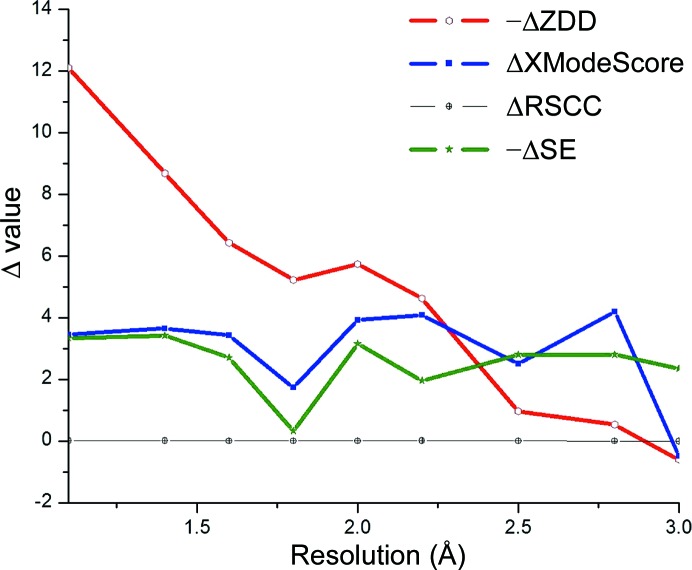
The differences in the raw values of ZDD, RSCC, strain energy (SE) and XModeScore for the AZM binding modes **3** and **2** plotted *versus* the resolution.

Through the truncation of the original high-resolution data set 3hs4, one can explore how well the method maintains its predictive power over decreasing resolution (Table 1 ▸) while controlling for inconsistencies in experimental conditions (pH, temperature, solvent and so on) between native high- and low-resolution structures. For the 3hs4 refinement, *XModeScore* is able to remain predictive until a low resolution of 3.0 Å is achieved. ΔZDD is the change in ZDD between one tautomer and another tautomer and is an indication of how well the ZDD will differentiate between the tautomers. At resolutions higher than 1.8 Å tautomer **2** exhibits a high value of ΔZDD. However, ΔZDD decreases towards zero when the resolution decreases to 3.0 Å as molecular details of the structure are becoming smeared, as discussed above. At a resolution of 2.8 Å ΔZDD is close to zero, which prevents a reliable distinction between forms **2** and **3** based on the density score alone. Generally speaking, ZDD tends to diminish in magnitude and equalize between tautomers at lower resolutions. On the other hand, when considering the overall ΔXModeScore, the value changes far less and is fairly flat, suggesting that even if the experimental density deteriorates with the resolution, the second component (*e.g.* ligand strain) significantly augments the deteriorating ΔZDD value and leads to the selection of the correct tautomer form at lower resolutions. It is notable that the ΔRSCC function is the flat, virtually zero line. This relationship underscores the fact that the RSCC undergoes very little change between modes. This observation is consistent with the conclusion above that RSCC is not likely to be an appropriate metric for scoring. For clarity, these relationships are plotted in Fig. 3 ▸.

### 3.3. AZM in PDB entry 4k0s at 1.8 Å resolution   

While using truncated data is an expedient and straightforward method of exploring predictability, a truncated high-resolution data set still has much better quality in terms of the merging *R*_merge_ factor of diffraction data, their completeness and redundancy, and of the mean signal-to-noise (*I*/σ) ratio when compared with those of the native low-resolution data (Wlodawer *et al.*, 2008 ▸). Therefore, we repeated our study on another structure of HCA II complexed with AZM (PDB entry 4k0s) determined at the more modest resolution of 1.8 Å. Again, just as in the 3hs4 case, *XModeScore* found that tautomer **3** is the preferable tautomer according to both ZDD and SE components (Supplementary Table S1).

### 3.4. The 8HX inhibitor in PDB entry 4n9s   

**Figure 4:**
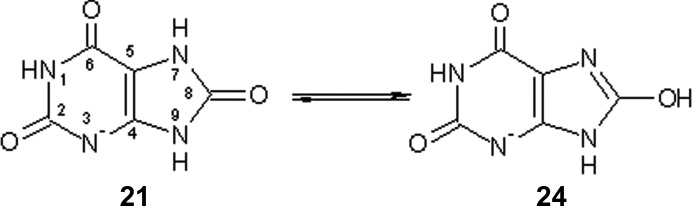
The key lactam–lactim equilibrium for the ligand 8HX (Oksanen *et al.*, 2014 ▸).

The enzyme urate oxidase is involved in the metabolism of purines, and to investigate the mechanism of action of urate oxidase the neutron diffraction structure of the enzyme in complex with uric acid monoanion (the inhibitor 8HX) was determined (Oksanen *et al.*, 2014 ▸; PDB entry 4n9m). In particular, the authors showed that the inhibitor is present in the form of the 8-hydroxyxanthine monoanion **24** that exists in equilibrium with form **21** in solution (Fig. 4 ▸).

The neutron diffraction structure 4n9m revealed that the form **24** occurs in the crystal. Such a conclusion is supported by an unambiguous deuterium density peak near the O atom at position 8, reflecting the hydroxyl group. The authors postulate that there is a water molecule near the hydroxyl O(8)H observed in the neutron diffraction experiment that might stabilize form **24**. However, a water molecule is capable of being both a donor and an acceptor of hydrogen bonds, and it is more likely to adapt to the solute (protein–ligand complex) rather than decisively determine its protonation state (Krieger *et al.*, 2012 ▸). Since this water molecule is not seen in the relevant high-resolution X-ray structure 4n9s, we believe that the hydrogen bond between N7 of the ligand and N—H (backbone) of Thr57 observed for the symmetry-related protein molecule in the crystal favors an unprotonated N7 and hence the tautomer **24**.

As many as 30 tautomer candidates of the two protonation states of 8HX were generated by *WashMoleculeMOE* (Supplementary Fig. S1), and *XModeScore* scores tautomer **24** at the top of the list based upon both scoring components (Supplementary Table S2). Comparing **24** (XModeScore 3.87) with its counterpart **21** (XModeScore 1.14) in the equilibrium shows the clear preference for the former. Additionally, the ZDD for **24** is lower (better) than the ZDD of **21** by 3.2 units. Truncating the resolution of the data, followed by QM refinement of the same set of tautomer structures, generally shows a similar trend until the low resolution of 3.0 Å is reached: the tautomer **24** remains at the the top of the list, while form **17** is consistently at the bottom (Supplementary Table S2).

### 3.5. The protonation state of the catalytic Asp215 in 2jjj   

**Figure 5:**
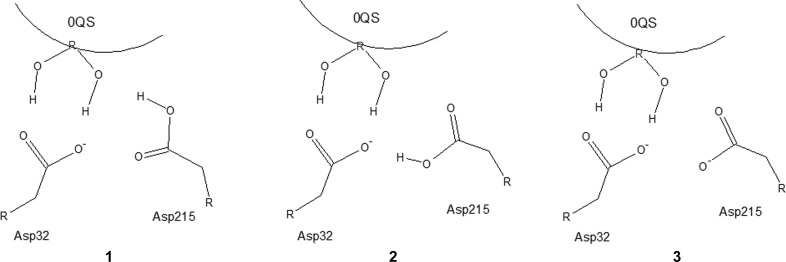
Three possible protonation states of Asp215 in the PD-135,040–aspartic proteinase structure.

Aspartic proteinases are enzymes that are involved in many metabolic processes and are associated with the progression of a number of diseases, including AIDS (Cooper, 2002 ▸; Davies, 1990 ▸); in recent years, aspartic proteinases have received significant attention as promising drug-design targets (Eder *et al.*, 2007 ▸). Several crystal structures of aspartic proteinases with a number of inhibitors are known, including the inhibitor PD-135,040 (PDB ligand 0QS; Supplementary Fig. S2), for which a neutron diffraction study has also been conducted (Coates *et al.*, 2008 ▸). The preliminary X-ray study (Coates *et al.*, 2003 ▸) demonstrated that the diol group of the ligand makes strong hydrogen bonds to two catalytic residues of the enzyme: Asp32 and Asp215. The neutron diffraction model (Coates *et al.*, 2008 ▸) revealed that the outer O atom of Asp215 is protonated (structure **1** in Figs. 5 ▸ and 6 ▸).

**Figure 6:**
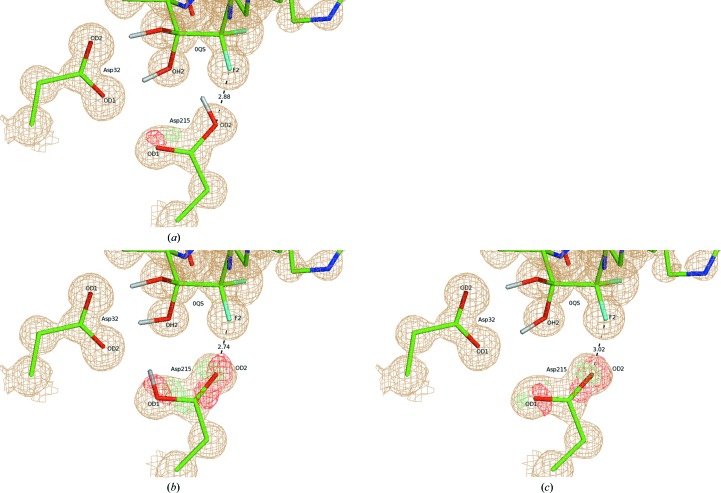
The catalytic center of aspartic proteinase with bound inhibitor 0QS in three alternative binding modes of Asp215, **1** (*a*), **2** (*b*) and **3** (*c*), after QM refinement of PDB entry 2jjj. The difference density around the side chain of the key catalytic residue Asp215 is contoured at the 3.5σ level. Only selected atoms of 0QS are shown for clarity.

**Figure 7:**
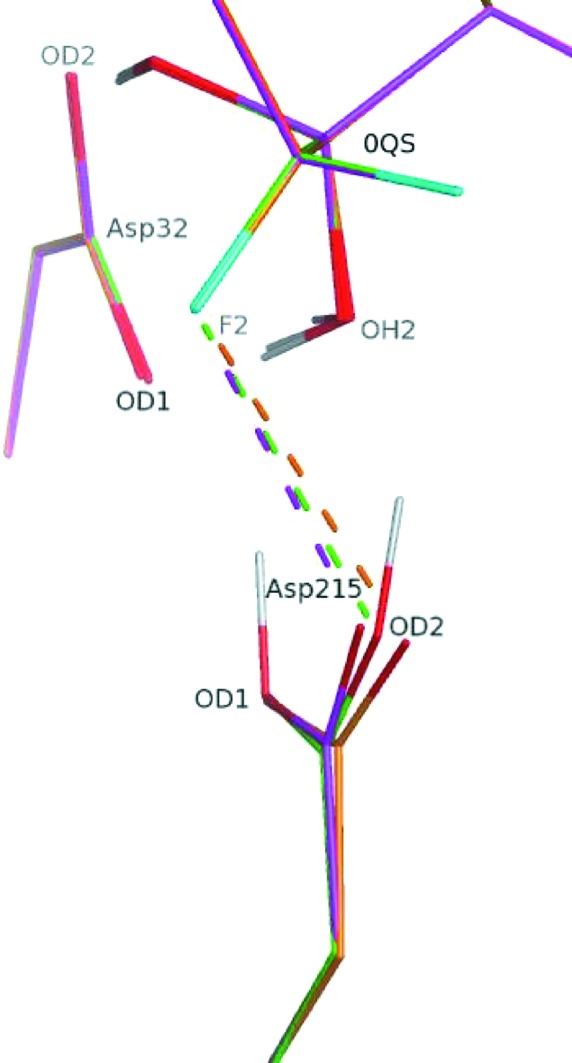
Superimposition of the key residues of the aspartic proteinase structure (PDB entry 2jjj) after QM refinements of three alternative binding modes of Asp215: **1** (green), **2** (magenta) and **3** (orange). Only selected atoms of 0QS are shown for clarity. The key distances are listed in Table 4 ▸.

For this case study, we generated the alternative structure **2** that has Asp215 protonated at the inner O atom, as well as structure **3** with a fully deprotonated Asp215 (Fig. 5 ▸). The *XModeScore* results for forms **1**–**3** after QM region refinement against the high-resolution X-ray structure 2jjj (Table 3 ▸) demonstrate an interesting interplay between the SE strain and ZDD components used within *XModeScore*. In this case, the SE strain of Asp215 rather than the ligand is considered as we vary the protonation states of the amino acid. In particular, protonation form **2** has the lowest strain energy, while the strain energy of the correct form **1** is about 3 kcal higher. Nevertheless, protonation form **1** of Asp215 is correctly scored as the best form owing to markedly better ZDD values. Such a low ZDD of form **1** can primarily be attributed to a positioning of the carboxyl group of Asp215 that is in much better agreement with experimental structure amplitudes compared with the other two protonation states. Indeed, difference density peaks around the carboxyl group are much lower for state **1** (Fig. 6 ▸). The location of the OD2 atom of Asp215 is particularly important. Its atomic ZDD in binding mode **1** is about fourfold better than that for states **2** and **3** (Table 4 ▸). The superimpositions of the atomic coordinates of Asp215 in all three forms after QM refinement (Fig. 7 ▸) revealed that OD2 in **1** is located in between the positions of this atom in the structures **2** and **3**, which is also strongly correlated with the distance Asp215 OD2–0QS F2. Indeed, while the separation between the F atom of the inhibitor and Asp215 OD2 in **1** is 2.88 Å, the same distance is greater for form **3** by 0.14 Å but is shorter for state **2** by 0.14 Å (Table 4 ▸). Thus, the protonated atom OD2 in **1** apparently adopts an optimal location and even a relatively small shift such as 0.14 Å in any direction, as seen in **2** and **3**, leads to a dramatic increase of the atomic ZDD owing to an increase in the disagreement with the experimental density (Fig. 7 ▸ and Table 4 ▸).

**Figure 8:**
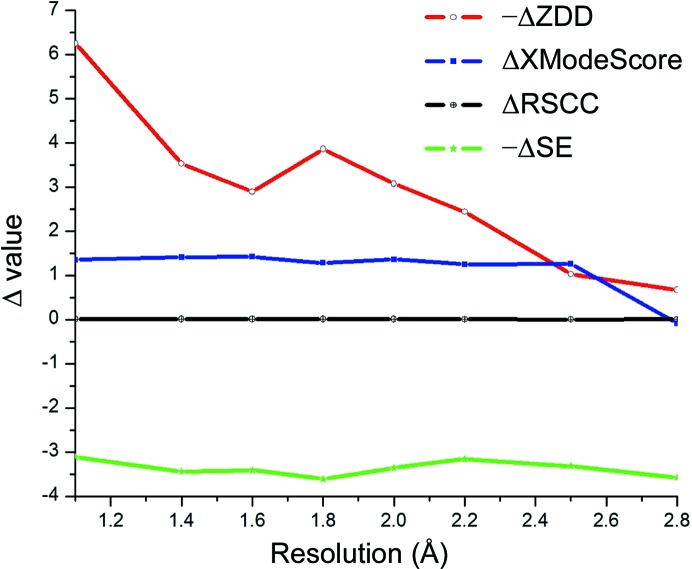
The differences in the raw values of ZDD, RSCC, strain energy (SE) and XModeScore for Asp215 binding modes **1** and **2** plotted *versus* resolution for PDB entry 2jjj.

At resolution truncations below 2.0 Å, the ZDD scores of forms **1** and **3** become similar. However, form **1** remains the top structure because its strain energy is lower than that of the unprotonated Asp215 form **3**. When the data are truncated, this relationship is maintained until the resolution reaches 2.8 Å and the scoring model no longer predicts the correct structure **1**. Overall, the plots of Δ values in Fig. 8 ▸ for the structure 2jjj look similar to the same plot for the AZM-binding modes (Fig. 3 ▸). In particular, the ΔZDD significantly decreases with resolution, while the ΔXModeScore function exhibits an essentially flat trend up to 2.8 Å resolution. Generally, the plots in Figs. 3 ▸ and 8 ▸ confirm the universal nature of the *XModeScore* concept.

## 4. Discussion   

In order to properly guide SBDD efforts, it is necessary to identify the correct tautomer/protomer state of the molecule in the bound state (Martin, 2009 ▸; Pospisil *et al.*, 2003 ▸). The building blocks of common drug and drug-candidate small molecules include 5,6-membered heterocycles and various functional groups that make proton migration from one part of the molecule to another possible. Prototropy or proton-shift tautomerism represents the most common type of molecular rearrangement relevant to SBDD. Keto–enol, imine–enamine and other equilibrium types lead to hydrogen transfer between hydrogen-donor groups (*e.g.* —OH, —NH2) and hydrogen-acceptor atoms (*e.g.* =O, =N—) (Warr, 2010 ▸). While the tautomerism changes neither the molecular formula nor the molecular charge, each tautomer is a distinct chemical structure with unique physico-chemical properties. The key point is that different tautomers exist in an equilibrium in solution where the ratio between possible states is affected by the pH, temperature, concentration, ionic strength and other factors (Raczyńska *et al.*, 2005 ▸). The general view is that protein receptors are capable of selectively binding a certain tautomeric form or forms from the mixture of several possible states (Pospisil *et al.*, 2003 ▸). For example, the antibiotic tetracycline can exist and react in one of 64 possible tautomeric forms adapting to various chemical environments (Duarte *et al.*, 1999 ▸). A growing body of evidence indicates that sometimes an unexpected tautomer form, or a form which does not correspond to the energy minimum of the tautomer set in vacuum, is found to react with the protein receptor (Martin, 2009 ▸).

The limitations of the current experimental techniques used for structure determination, where even at the extremely high resolution of 0.66 Å only 54% of all H atoms are revealed (Howard *et al.*, 2004 ▸), make it difficult to determine these states. As an alternative to X-ray crystallography, neutron diffraction is considered to be a unique technique that allows experimental determination of hydrogen positions in crystal structures at resolutions much lower than those used to reveal atomic details (Blum *et al.*, 2009 ▸; Katz *et al.*, 2006 ▸). However, owing to the limitations of neutron diffraction such as a reliance on large crystals, the necessity of deuterium exchange, the limited availability of sources of neutron radiation and difficulties in the refinement of H atoms with negative scattering length, neutron diffraction is of only limited utility in SBDD. In fact, it is notable that, as of June 2015, the overall number of structures determined using neutron diffraction available in the PDB remains at 88 *versus* the total of 97 297 X-ray structures.

We have found that *XModeScore* is able to determine the protonation state of ligands and catalytic residues using routine X-ray crystallographic data with a level of accuracy that is only achieved in neutron diffraction studies coupled with high-resolution X-ray structures. Even when *XModeScore* is challenged with truncated or low-resolution (*e.g.* 2.5–3.0 Å) X-ray data, it is still observed to be predictive. The *XModeScore* method involves the QM X-ray refinement of a set of macromolecular structures containing all likely tautomer/protomer forms or binding modes, followed by a rigorous statistical analysis of difference electron-density maps around each candidate form coupled with computation of its QM strain. This approach allows us to choose the best tautomer based on a combination of energetics and of agreement between model and experimental density. Yu *et al.* (2006 ▸) have proposed a similar setup when a set of several protein structures with different protonation states of three key residues is refined with the *CNS* package using the QM/MM protocol as described elsewhere (Yu *et al.*, 2005 ▸). After each refinement, the relative stabilities of these protonation states were evaluated from thermodynamic cycles using energies from additional single-point semiempirical *DivCon* calculations. The key advantage of *XModeScore* over the above procedure (Yu *et al.*, 2006 ▸) is that it directly employs the experimental electron density to judge the bound protomer.

In order to validate the applicability of *XModeScore* in the present work, we considered several key case studies. As the first example, for many years the correct binding form of the drug acetazolamide (AZM) in human carbonic anhydrase II was uncertain (Lesburg *et al.*, 1997 ▸; Sippel *et al.*, 2009 ▸) and the correct form was only unambiguously established by a rigorous joint neutron diffraction/X-ray study in 2012 (Fisher *et al.*, 2012 ▸). With *XModeScore*, the same conclusion was reached utilizing the X-ray data alone and it chose the correct tautomeric form over two other possible states of AZM by a wide margin (Table 1 ▸). At the structural level, the difference between the correct form **3** and the incorrect binding modes is primarily attributed to shortening of the Zn—N coordination bond between the N atom of the AZM sulfonamido group and the cofactor of the enzyme seen in structure **3** (Table 2 ▸) after QM refinement. Notably, the Zn—N distance of 1.9 Å in **3** is shorter than the average distance of 2.00 Å for the Zn—N bond type (Harding, 1999 ▸) that is typically used for link-restraint parameters in conventional refinement, suggesting that without *a priori* knowledge of the correct outcome it would be difficult for conventional, restraint-based refinement to come to the same conclusion. Nevertheless, such a short Zn—N distance gives rise to the best agreement with the experimental data observed for binding mode **3** (Fig. 2 ▸ and Tables 1 ▸ and 2 ▸). This example underscores the importance of QM refinement as the indispensable step in successful scoring of the tautomer/protomer set. The key and unique advantage of QM refinement is to derive the geometry of protein–ligand systems objectively without making any *a priori* assumptions in the form of CIF dictionaries, fixed atom types, link restraints, coordination-sphere parameters or other ‘user-supplied’ characteristics (Borbulevych *et al.*, 2014 ▸). When considering conventional refinement (Supplementary Tables S7 and S8) for 3hs4 mode **3** is still shown to be the best structure; however, the greater sensitivity of the QM-based refinement is apparent when one considers the spread of the ZDD score and XModeScore values across the three modes. In each case the ‘spread’ or discriminatory power of these indicators is much higher for the QM-based refinement. This is a crucial difference from scoring based on QM refinement, which demonstrates that QM-based refinement is better able to discriminate the correct mode **3** by a wide margin in both ZDD score and XModeScore, as was discussed above. One could speculate that this result could be owing to the fact that while the *eLBOW*-generated CIF for each tautomer captures the intramolecular conformational changes associated with protonation-state changes, the intermolecular interactions (*e.g.* electrostatics, polarization, charge transfer and so on) are not captured in the conventional refinement, and hence any impact of the active site on the different ligand protonation states are likewise missing. To further reinforce this point, we conducted another round of conventional refinement with AZM restraint CIFs provided by *Mogul*, which are based on small-molecule crystallographic data from the Cambridge Structural Database (CSD; Supplementary Table S8). Clearly, *Mogul* restraints lead to lower strain energies of AZM, but the scoring results are essentially similar to those obtained with *eLBOW* restraints. Notably, the conventional refinements resulting from either type of CIF ceased to predict the correct tautomer after even a modest truncation of the resolution to 1.6 Å (Supplementary Tables S7 and S8). This result shows that even high-quality stereochemical restraints cannot overcome the deficiency of the energy function used in conventional refinement, underscoring the superiority of QM refinement for *XModeScore*. Finally, for the sake of completeness, we also validated *XModeScore* with the AM1 Hamiltonian (Dewar *et al.*, 1985 ▸; Supplementary Tables S3 and S6) and found that the scoring results are essentially similar to those with the PM6 Hamiltonian.

A large proportion of the available neutron diffraction experiments are focused on studies of enzymatic mechanisms in order to establish protonation states and the hydrogen-bond network within the enzyme catalytic center (Blum *et al.*, 2009 ▸; Tomanicek *et al.*, 2013 ▸). The aspartic proteinase case study (Tables 3 ▸ and 4 ▸ and Figs. 5 ▸ and 6 ▸) clearly demonstrates that *XModeScore* is able to efficiently investigate the protonation state of the key catalytic residue Asp215 using X-ray data alone and to ultimately select the state which corresponds to that found in the neutron diffraction study reported by Coates *et al.* (2008 ▸). In this case, we discovered that there is a strong correlation between the distance Asp215 OD2–0QS F2 and the size of the difference density peaks or the magnitude of ZDD around the Asp215 OD2 atom (Table 4 ▸ and Fig. 7 ▸). A review (Müller *et al.*, 2007 ▸) underscores the importance of fluorine substituents in SBDD since fluorine has unique properties which impact ligand affinity owing to polar hydrophobicity (Biffinger *et al.*, 2004 ▸). The ability of fluorine to modulate ligand binding and even the immune response in peptide-based immunotherapy has been well documented (Gómez-Nuñez *et al.*, 2008 ▸; Müller *et al.*, 2007 ▸; Piepenbrink *et al.*, 2009 ▸). Coates *et al.* (2008 ▸) do not elaborate on the possible fluorine effect in their manuscript; however, our results suggest that the protonation of Asp215 in the structure with the inhibitor 0QS (PD-135,040) might be modulated by the neighboring F atom rather than generally represent the mechanism of action of aspartic proteinase. Again, as in the AZM case, when considering the conventional refinement results provided in Supplementary Tables S9 and S10, the ‘spread’ of the XModeScore and ZDD score of the three Asp215 tautomers is an order of magnitude greater and the residue structural strain values are an order of magnitude lower for the QM-based refined models, suggesting that the QM values are likely to be more robust. This said, it is interesting to note that the ZDD of mode **1** is lower (better) than the ZDD of mode **1** observed in the QM-based refinement. Upon further exploration, the elevated ZDD score is associated with the backbone O and C atoms of Asp215, which can be attributed to the fact that SE methods such as PM6 overestimate the lengths of some protein backbone bonds (Borbulevych *et al.*, 2014 ▸; Yu *et al.*, 2005 ▸)

In the case study of the urate anion (ligand 8HX; Supplementary Table S2), *XModeScore*, using QM-based refinement, is able to select the correct tautomer from a large number of possible states using the very wide range of data resolution between 1.0 and 3.0 Å. Given the large number of possible tautomeric forms, it is interesting to consider the p*K*_a_ of the structure. Uric acid has two p*K*_a_ values (5.4 and 9.8) that are relevant in the physiological pH range considered (Simic & Jovanovic, 1989 ▸). As a result, this compound exists predominantly as a monoanionic form. However, this monoanion can undergo lactam–lactim tautomerism as shown in Fig. 4 ▸ and it can exist in a number of other anionic tautomeric forms (Supplementary Scheme S1). Therefore, the p*K*_a_ values alone do not allow us to determine the correct tautomer form because all tautomers have the same number of H atoms and the same molecular charge (Haranczyk *et al.*, 2008 ▸). When considering conventional refinement, as shown in Supplementary Tables S4 and S5, while the large number of possible tautomers did allow conventional refinement to yield XModeScores and ZDD scores with comparable standard deviations, the conventional refinement was unable to determine the correct tautomer.

## 5. Conclusions   

With the calculations performed to date involving protomer/tautomer-state determination, *XModeScore* has shown itself to be versatile and robust. Further, while the method could be used with either QM-based refinement or conventional refinement, the significance of the QM-based results clearly appears to be noticeably higher than that observed in conventional refinement even when advanced types of ligand restraints (*e.g.* *Mogul* CIF) are employed. Another related area of interest is in the exploration of heavy-atom flip-state ambiguity often observed in macromolecular X-ray crystallo­graphy. X-ray studies of protein–ligand complexes reliably reveal only the configuration of heavy atoms of the structure, with the caveat that elements with similar atomic numbers, such as N and O, are often indistinguishable at modest resolutions. This leads to ambiguous orientations of molecule fragments capable of flipping, such as imidazole rings, amide groups and so on. Serious challenges in assigning the correct ligand orientation/flipping in X-ray macromolecular crystallography have been well documented and recognized (Malde & Mark, 2011 ▸). Often, the hypothetical flip state is chosen based upon its agreement with the hydrogen-bond network and van der Waals contacts with the residue in question (Word *et al.*, 1999 ▸). Not only does our method offer an entirely new X-ray data-driven approach for selecting flip states, but broadly speaking, any docking/placement of a ligand within the ‘blob’ of electron density can be addressed using our method. Further studies of this phenomenon will be explored in subsequent work.

## Supplementary Material

Supporting Information. . DOI: 10.1107/S2059798316002837/rr5117sup1.pdf

## Figures and Tables

**Table 1:** Scoring results for possible tautomeric structures of the ligand AZM in PDB entry 3hs4 The correct tautomeric state data are highlighted in bold.

	SE	RSCC	ZDD	XModeScore
Structure 3hs4				
**3**	**5.55**	**0.989**	**12.8**	**2.72**
2	8.89	0.978	24.9	−0.74
1	10.8	0.975	27.2	−1.98
Resolution 1.4 Å				
**3**	**5.89**	**0.989**	**9.42**	**2.77**
2	9.31	0.981	18.1	−0.88
1	10.1	0.978	20.9	−1.88
Resolution 1.6 Å				
**3**	**6.01**	**0.987**	**7.87**	**2.72**
2	8.71	0.980	14.3	−0.70
1	9.75	0.978	16.8	−2.02
Resolution 1.8 Å				
**3**	**6.13**	**0.988**	**6.18**	**2.13**
2	6.46	0.982	11.4	0.40
1	9.24	0.978	14.6	−2.53
Resolution 2.0 Å				
**3**	**5.58**	**0.989**	**6.56**	**2.68**
2	8.74	0.982	12.3	−1.24
1	7.86	0.975	15.6	−1.45
Resolution 2.2 Å				
**3**	**5.77**	**0.989**	**6.17**	**2.77 **
2	7.73	0.981	10.8	−1.31
1	8.35	0.984	10.0	−1.47
Resolution 2.5 Å				
**3**	**5.40**	**0.989**	**7.65**	**2.47**
2	8.20	0.986	8.62	−0.04
1	11.1	0.984	9.48	−2.43
Resolution 2.8 Å				
**3**	**5.45**	**0.984**	**9.67**	**2.80**
2	8.25	0.984	10.2	−1.39
1	8.74	0.982	10.1	−1.41
Resolution 3.0 Å				
2	8.02	0.983	11.3	0.49
**3**	**5.67**	**0.981**	**11.9**	**0.01**
1	8.61	0.983	11.5	−0.50

**Table 2:** Geometry and electron-density characteristics (ZDD−/ZDD+) of the N1 atom of AZM directly bound to the Zn atom in the structure with PDB code 3hs4 The correct tautomeric state data are highlighted in bold.

3hs4 AZM tautomers	1	2	**3**
Zn–AZM N1 (Å)	2.05	2.07	**1.90**
AZM N1 ZDD−/ZDD+	14.27/20.45	14.58/21.64	**4.35**/**4.66**

**Table 3:** Scoring results for possible protonated states of Asp215 in PDB entry 2jjj The correct protonated state data are highlighted in bold.

	SE	RSCC	ZDD	XModeScore
Structure 2jjj				
**1**	**12.2**	**0.987**	**6.05**	**1.59**
2	9.08	0.977	12.3	0.24
3	17.3	0.979	11.3	−1.83
Resolution 1.4 Å				
**1**	**12.7**	**0.990**	**3.66**	**1.33**
2	9.26	0.983	7.19	−0.08
3	17.3	0.987	5.36	−1.25
Resolution 1.6 Å				
**1**	**12.7**	**0.988**	**3.26**	**1.43**
2	9.29	0.980	6.15	0.01
3	17.5	0.983	4.98	−1.44
Resolution 1.8 Å				
**1**	**12.9**	**0.989**	**2.66**	**1.09**
2	9.29	0.981	6.52	−0.19
3	17.5	0.987	3.68	−0.90
Resolution 2.0 Å				
**1**	**12.6**	**0.989**	**1.87**	**1.13**
2	9.24	0.982	4.94	−0.23
3	17.5	0.987	2.64	−0.90
Resolution 2.2 Å				
**1**	**12.4**	**0.986**	**2.92**	**0.97**
2	9.24	0.981	5.36	−0.28
3	17.4	0.987	3.10	−0.68
Resolution 2.5 Å				
**1**	**12.8**	**0.988**	**1.35**	**0.98**
2	9.48	0.986	2.38	−0.28
3	17.9	0.989	1.46	−0.71
Resolution 2.8 Å				
2	9.52	0.987	1.90	0.03
3	17.9	0.991	0.186	0.02
**1**	**13.1**	**0.99**	**1.23**	**−0.06**

**Table 4:** Selected interatomic distances and electron-density characteristics (atomic ZDD−/ZDD+) for atoms of the catalytic residue Asp215 in PDB entry 2jjj The correct protonated state data are highlighted in bold.

2jjj Asp215 states	**1**	**2**	**3**
Asp215 OD2–0QS F2 (Å)	**2.88**	2.74	3.02
Asp215 OD2–0QS OH2 (Å)	**2.65**	2.64	2.73
Asp215 OD1–Asp32 OH2 (Å)	**2.90**	2.88	3.00
Asp215 OD1: ZDD−/ZDD+	**5.98**/**3.76**	7.20/5.09	7.78/5.35
Asp215 OD2: ZDD−/ZDD+	**3.66**/**1.23**	10.96/7.79	12.89/8.10
