# Supplementary material for: XModeScore: a novel method for accurate protonation/tautomer-state determination using quantum-mechanically driven macromolecular X-ray crystallographic refinement
Source: Acta Crystallogr D Struct Biol. 2016 Mar 30;72(Pt 4):586–98. doi: 10.1107/S2059798316002837 (PMC4822566; doi:10.1107/S2059798316002837)
Supplement: Supplementary file 1 [file d-72-00586-sup1.pdf]

STRUCTURAL
BIOLOGY

Volume 72 (2016)

Supporting information for article:

XModeScore: a novel method for accurate protonation/tautomer-state determination using quantum-mechanically driven macromolecular X-ray crystallographic refinement

Oleg Borbulevych, Roger I. Martin, Ian J. Tickle and Lance M. Westerhoff

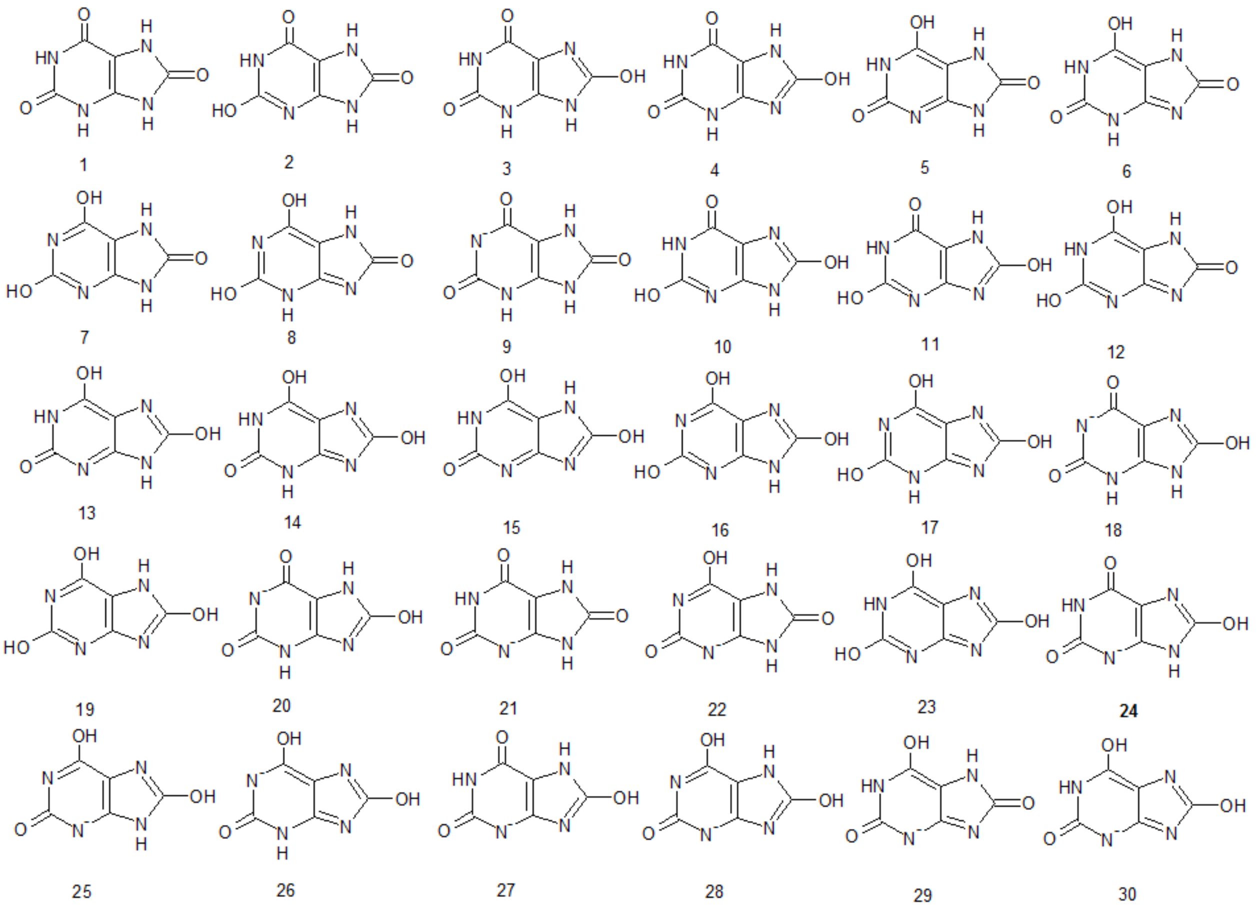

Figure S1 Possible tautomeric/protonation states of the ligand 8HX included in XModeScore scoring.

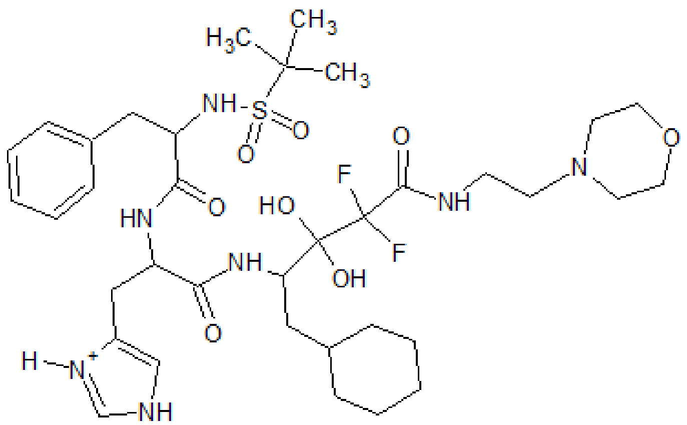

PD-135.040 (PDB ligand 0QS)

Figure S2 Structure of the inhibitor PD-135040 (PDB ligand 0QS).

Table S1 Scoring results for possible tautomeric structures of the ligand AZM in PDB 4K0S

Structure- 4K0S	SE	RSCC	ZDD	XModeScore
**3**	**5.16**	**0.987**	**4.88**	**2.75**
2	8.95	0.985	5.88	-0.82
1	9.69	0.984	6.33	-1.93
**Resolution 2.0 Å**				
**3**	**5.23**	**0.988**	**3.74**	**2.78**
2	8.91	0.985	4.99	-0.93
1	9.61	0.984	5.36	-1.84
**Resolution 2.2 Å**				
**3**	**5.24**	**0.991**	**2.51**	**2.76**
2	8.73	0.988	4.11	-0.96
1	10.3	0.989	4.18	-1.8
**Resolution 2.5 Å**				
**3**	**5.27**	**0.993**	**3.21**	**2.47**
2	10.4	0.991	3.66	-0.87
1	8.76	0.99	4.38	-1.6
**Resolution 2.8 Å**				
**3**	**5.2**	**0.99**	**4.97**	**1.07**
1	9.59	0.989	3.86	0.36
2	8.47	0.985	5.48	-1.43
**Resolution 3.0 Å**				
**3**	**5.11**	**0.992**	**5.18**	**1.77**
1	10.4	0.99	4.98	-0.20
2	8.34	0.986	5.98	-1.57

Table S2 Scoring results for possible tautomeric structures of the ligand 8HX in PDB 4N9S based on the QM refinement with PM6.

Structure-4N9S	SE	RSCC	ZDD	XModeScore
**24**	**4.42**	**0.961**	**16.1**	**3.87**
25	7.01	0.958	18.1	2.11
18	9.84	0.958	18.1	2.02
20	5.76	0.958	18.3	2.01
28	5.14	0.959	18.3	2.01
22	3.76	0.959	18.3	2.00
30	5.01	0.959	18.3	1.95
26	7.5	0.958	18.6	1.66
9	5.48	0.958	19.2	1.19
21	5.19	0.958	19.3	1.14
14	19.2	0.958	19.5	0.68
13	12.9	0.958	19.7	0.62
27	3.99	0.954	20.2	0.45
6	25.9	0.959	19.8	0.28
5	10.8	0.956	20.6	-0.06
3	74.3	0.959	19.2	-0.19
15	14.7	0.953	21	-0.46
7	95.9	0.96	19	-0.49
29	12.8	0.955	21.3	-0.70
10	112	0.96	19.1	-0.84
1	91.7	0.96	19.7	-0.99
19	123	0.957	19.6	-1.56
23	114	0.96	19.9	-1.63
4	115	0.957	20	-1.72
8	119	0.958	20	-1.74
11	104	0.955	20.6	-1.97
2	108	0.958	20.8	-2.17
16	117	0.956	20.5	-2.19
12	107	0.956	20.9	-2.25
17	100	0.951	22	-3.04
**Resolution 1.4 Å**				
**24**	**4.81**	**0.967**	**11.9**	**3.82**
20	5.64	0.965	13.9	2.09
25	6.87	0.963	14	2.03
28	5.23	0.963	14.1	1.93
22	4.72	0.962	14.2	1.88
30	5.06	0.963	14.2	1.87
26	7.61	0.963	14.3	1.70
18	10.6	0.962	14.3	1.68
21	4.47	0.963	14.6	1.51

9	6	0.962	15	1.17
13	13.3	0.961	15.4	0.65
27	4.71	0.958	15.8	0.51
14	19.1	0.962	15.6	0.37
15	12.6	0.959	15.9	0.27
6	27.3	0.963	15.8	0.07
5	10.5	0.96	16.4	-0.10
3	79.4	0.963	15.3	-0.58
7	95.6	0.965	14.9	-0.60
1	91.2	0.966	15.2	-0.72
10	112	0.964	15	-1.01
29	13	0.957	17.6	-1.20
4	115	0.963	15.3	-1.29
19	124	0.963	15.3	-1.51
23	114	0.963	15.6	-1.57
16	117	0.962	15.6	-1.61
2	107	0.964	15.9	-1.68
11	92	0.96	16.5	-1.86
8	119	0.962	16.2	-2.13
12	113	0.961	16.4	-2.22
17	100	0.954	18.2	-3.47

Resolution 1.6 Å

**24**	**5.06**	**0.969**	**10**	**3.60**
18	10.2	0.966	11.6	2.25
21	5.2	0.966	12.1	2.01
20	5.42	0.967	12.1	1.99
25	8.38	0.965	12	1.99
30	4.89	0.964	12.5	1.68
9	5.11	0.967	12.6	1.63
28	5.19	0.965	12.8	1.42
22	4.64	0.962	12.9	1.36
26	6.17	0.964	13.1	1.24
13	13.4	0.963	13.4	0.79
27	4.37	0.961	13.9	0.60
14	18.9	0.964	13.6	0.57
15	14.4	0.96	14	0.35
6	25.5	0.963	13.7	0.32
3	78.1	0.967	12.9	-0.12
5	10.4	0.959	14.9	-0.28
10	112	0.968	12.3	-0.37
1	91.9	0.967	13.3	-0.74
29	13.3	0.957	15.7	-0.95
7	96.5	0.967	13.6	-1.03
4	115	0.966	13.3	-1.19
2	108	0.965	14.1	-1.65
11	92.1	0.963	14.7	-1.78

23	113	0.964	14.1	-1.80
16	117	0.964	14.1	-1.89
12	107	0.963	14.5	-1.97
8	117	0.963	14.5	-2.17
19	122	0.964	14.4	-2.23
17	100	0.957	16.8	-3.64
**Resolution 1.8 Å**				
**24**	**4.9**	**0.966**	**8.5**	**2.64**
21	4.78	0.966	8.76	2.40
20	5.12	0.967	8.99	2.19
22	3.87	0.962	9.14	2.08
28	4.46	0.962	9.71	1.55
9	5.52	0.964	9.87	1.39
27	4.54	0.961	9.91	1.37
30	4.09	0.96	10	1.28
15	13.7	0.961	10.2	0.96
6	26.9	0.964	9.93	0.92
18	9.5	0.96	10.4	0.83
5	10.5	0.96	10.4	0.81
25	7.5	0.958	10.6	0.70
13	13.1	0.96	10.6	0.56
26	6.06	0.958	11	0.37
1	92.3	0.966	9.26	0.27
14	17.2	0.961	11.2	0.00
4	116	0.966	9.39	-0.31
3	86.6	0.964	10	-0.32
7	96.1	0.965	9.82	-0.32
29	13.4	0.958	11.9	-0.61
2	107	0.965	10.4	-1.04
11	93	0.964	10.8	-1.14
10	112	0.963	10.5	-1.24
19	122	0.962	10.6	-1.50
12	115	0.962	10.8	-1.60
8	120	0.962	11.2	-2.03
23	113	0.96	11.4	-2.12
16	118	0.957	12	-2.71
17	151	0.951	14.2	-5.37
**Resolution 2.0 Å**				
**24**	**3.96**	**0.972**	**6.47**	**2.87**
21	4.39	0.97	6.99	2.40
20	4.93	0.972	7.22	2.19
9	5.31	0.969	7.74	1.73
27	4.03	0.969	7.97	1.55
28	5.05	0.968	8.08	1.43
22	4.89	0.966	8.14	1.39

30	4.18	0.966	8.39	1.18
18	9.42	0.965	8.29	1.16
6	24.7	0.969	8.02	1.11
25	7.14	0.962	8.84	0.73
15	13.4	0.966	8.81	0.64
1	92.4	0.972	7.18	0.55
29	13.4	0.965	8.92	0.54
5	10.5	0.964	9.09	0.44
26	5.59	0.962	9.5	0.18
13	13	0.963	9.52	0.02
3	88.3	0.969	7.91	-0.02
14	15.5	0.963	9.55	-0.06
4	116	0.971	7.6	-0.29
7	95.7	0.968	8.57	-0.73
2	108	0.969	8.42	-0.84
11	106	0.967	8.95	-1.26
10	112	0.966	8.83	-1.27
8	120	0.964	9.31	-1.85
12	108	0.964	9.56	-1.85
19	121	0.966	9.34	-1.90
23	114	0.963	9.9	-2.24
16	118	0.961	10.1	-2.54
17	151	0.955	12.5	-5.26

Resolution 2.2 Å

**24**	**4.04**	**0.981**	**4.85**	**3.44**
21	6.48	0.977	6.58	1.98
30	4.52	0.975	7.16	1.55
20	4.93	0.978	7.21	1.50
27	3.94	0.976	7.43	1.34
28	4.33	0.975	7.74	1.08
13	12.9	0.975	7.56	1.06
25	7.08	0.972	7.72	1.04
18	8.46	0.975	7.71	1.03
9	5.53	0.975	7.79	1.01
14	19.3	0.976	7.5	0.98
5	10.8	0.972	7.72	0.97
15	13.6	0.975	7.78	0.87
6	28.8	0.975	7.71	0.63
3	94.8	0.978	6.22	0.57
26	4.77	0.972	8.55	0.41
22	6.52	0.971	8.58	0.35
1	91.7	0.977	6.92	0.06
10	111	0.976	7.19	-0.54
2	109	0.976	7.37	-0.64
4	116	0.976	7.28	-0.71
7	94.9	0.974	7.83	-0.75

11	93.5	0.974	8.05	-0.90
29	13.6	0.968	10.1	-1.00
12	109	0.972	8.57	-1.61
23	114	0.972	8.67	-1.79
19	122	0.972	8.86	-2.11
8	123	0.971	8.91	-2.17
16	118	0.971	9.37	-2.44
17	151	0.968	12	-5.21

Resolution 2.5 Å

**24**	**3.81**	**0.988**	**4.54**	**2.70**
21	4.52	0.984	5.1	2.16
30	4.6	0.986	5.48	1.80
18	9.43	0.985	5.51	1.68
14	22.4	0.986	5.75	1.20
27	4.64	0.984	6.16	1.16
26	5.1	0.984	6.16	1.15
20	4.92	0.984	6.2	1.12
13	12.6	0.985	6.05	1.11
15	12.3	0.984	6.12	1.05
6	31.8	0.985	5.79	0.98
9	5.17	0.982	6.39	0.94
25	6.98	0.983	6.42	0.88
3	94.1	0.986	4.76	0.72
23	17.7	0.983	6.4	0.68
28	4.64	0.984	6.74	0.62
5	11	0.983	6.78	0.46
29	13.4	0.98	6.84	0.36
1	90.4	0.984	5.29	0.29
22	6.34	0.979	7.57	-0.19
10	113	0.984	6.15	-0.96
4	114	0.984	6.39	-1.19
2	107	0.983	6.53	-1.20
8	122	0.983	6.54	-1.51
11	92.8	0.984	7.19	-1.53
7	94.3	0.982	7.21	-1.59
12	110	0.983	7.56	-2.23
16	118	0.982	7.7	-2.51
19	123	0.983	7.75	-2.67
17	150	0.979	10.2	-5.48

Resolution 2.8 Å

**24**	**4**	**0.99**	**2.73**	**3.01**
30	4.99	0.986	3.61	2.07
26	5.1	0.984	3.75	1.93
14	27.8	0.983	3.53	1.72

13	12.3	0.984	4.09	1.44
25	6.79	0.983	4.21	1.42
15	13.3	0.986	4.22	1.29
18	8.63	0.983	4.35	1.25
21	4.61	0.982	4.64	1.02
6	33.5	0.983	4.11	1.01
27	3.73	0.985	5.16	0.50
5	10.3	0.983	5.2	0.33
28	4.05	0.982	5.48	0.17
9	4.97	0.981	5.48	0.14
22	3.19	0.978	5.8	-0.15
3	92.4	0.983	4.15	-0.17
10	113	0.985	3.77	-0.19
23	104	0.984	4.05	-0.30
20	5.01	0.983	5.92	-0.32
7	96.4	0.981	4.49	-0.60
2	108	0.984	4.35	-0.68
12	110	0.984	4.33	-0.69
29	12.9	0.98	6.18	-0.73
16	117	0.983	4.26	-0.75
1	90.6	0.982	4.92	-0.94
11	105	0.985	5.01	-1.31
19	124	0.983	4.78	-1.44
4	114	0.985	5.5	-1.99
8	121	0.981	5.41	-2.03
17	150	0.978	7.77	-5.03

Resolution 3.0 Å

**24**	**3.71**	**0.988**	**2.49**	**3.19**
13	12.2	0.985	3.42	2.09
5	9.62	0.985	3.52	2.04
26	5.63	0.985	3.74	1.89
30	4.99	0.985	3.82	1.82
14	32	0.986	3.39	1.74
15	13.5	0.987	3.9	1.58
29	4.16	0.985	4.16	1.50
18	9.38	0.983	4.1	1.45
25	7.02	0.983	4.31	1.29
28	4.04	0.987	4.61	1.04
22	3.3	0.982	4.88	0.80
6	35.2	0.986	4.39	0.66
21	4.73	0.981	4.99	0.65
9	5.28	0.982	5.25	0.38
20	4.9	0.985	5.54	0.10

27	3.89	0.985	5.57	0.09
3	93.8	0.983	4.06	-0.13
7	96.6	0.984	4.81	-0.94
10	112	0.983	4.58	-1.00
8	122	0.986	4.56	-1.17
23	114	0.984	4.98	-1.44
1	90.5	0.982	5.44	-1.45
12	110	0.985	5.21	-1.59
19	121	0.986	5	-1.61
16	118	0.983	5.08	-1.62
2	109	0.982	5.43	-1.79
11	103	0.985	5.95	-2.20
4	113	0.985	5.94	-2.39
17	149	0.979	7.82	-4.97

Table S3 Scoring results for possible tautomeric structures of the ligand 8HX in PDB 4N9S based on the **QM** refinement with AM1.

Structure-4N9S	SE	RSCC	ZDD	XModeScore
**24**	**5.44**	**0.962**	**16**	**3.74**
22	3.28	0.960	17.9	2.01
29	5.7	0.960	18.6	1.22
28	3.07	0.954	18.8	1.14
21	3.87	0.959	18.8	1.06
26	4.75	0.957	18.9	0.93
30	2.98	0.956	19	0.90
6	8.55	0.962	18.9	0.86
25	9.86	0.955	18.8	0.84
14	5.2	0.960	19	0.82
20	5.19	0.951	19.1	0.70
18	6.44	0.953	19.2	0.57
13	7.97	0.959	19.2	0.53
7	7.25	0.960	19.3	0.46
9	6.41	0.955	19.4	0.42
16	9.44	0.959	19.3	0.36
19	7.89	0.957	19.6	0.15
5	8.85	0.960	19.6	0.11
3	7.61	0.958	19.7	0.09
1	7.36	0.960	19.7	0.02
4	6.06	0.956	20.1	-0.24
27	3.5	0.952	20.2	-0.32
10	10.3	0.958	20	-0.32
17	13	0.956	20.1	-0.50
15	9.06	0.956	20.2	-0.51
8	8.97	0.954	21.3	-1.56
2	12.9	0.957	21.3	-1.71
23	87.8	0.958	19.5	-2.83
12	98.2	0.959	20.3	-4.00
11	93.7	0.952	21.4	-4.93

Table S4 Scoring results for possible tautomeric structures of the ligand 8HX in PDB 4N9S based on the **conventional** (no QM) Phenix refinement with eLBOW AM1 CIF.

Structure-4N9S	SE	RSCC	ZDD	XModeScore
25	14.8	0.960	17.4	2.55
20	10.7	0.957	18	2.52
22	22.1	0.964	16.4	2.32
**24**	**19.5**	**0.959**	**17.2**	**1.98**
30	14.9	0.959	18.3	1.67
26	14.4	0.958	18.5	1.56
1	12.5	0.964	18.9	1.48
21	20.6	0.962	17.7	1.31
18	16.7	0.959	18.5	1.18
5	23.4	0.966	17.4	1.17
14	12.3	0.962	19.4	1.01
23	8.27	0.957	20.3	0.74
16	20.2	0.961	18.9	0.32
3	21.8	0.963	18.9	0.02
29	29.8	0.963	17.8	-0.14
13	21.5	0.959	19.1	-0.15
27	19.4	0.955	19.7	-0.32
9	25.1	0.961	18.8	-0.39
7	27.7	0.964	18.7	-0.73
10	23.1	0.962	19.6	-0.83
17	21.2	0.960	19.9	-0.85
4	21	0.960	20.1	-0.93
28	26.3	0.955	19.3	-0.99
6	29	0.965	19.1	-1.26
2	26.9	0.964	19.5	-1.29
15	22.8	0.957	20.3	-1.40
12	30.4	0.964	19.8	-2.15
8	35.1	0.965	19.2	-2.32
11	29.5	0.958	20.9	-3.02
19	31.2	0.957	20.7	-3.05

Table S5 Scoring results for possible tautomeric structures of the ligand 8HX in PDB 4N9S based on the **conventional** (no QM) Phenix refinement with **Mogul** CIF.

Structure-4N9S	SE	RSCC	ZDD	XModeScore
26	17.7	0.967	16.9	4.60
22	17.3	0.965	20.6	2.39
25	17.3	0.962	20	2.78
21	17.8	0.965	21.4	1.85
**24**	**14.9**	**0.96**	**22.5**	**1.60**
28	20.1	0.959	22.2	1.01
9	23	0.965	22.1	0.63
5	24.4	0.967	21.3	0.95
30	23.2	0.963	21.7	0.89
27	18.3	0.959	23.1	0.72
2	23.3	0.968	23	0.06
14	17.3	0.963	23.4	0.66
1	22.2	0.967	23.2	0.11
17	21.6	0.964	23.2	0.20
23	23.7	0.964	23	0.03
20	24.1	0.96	23.7	-0.46
18	32.3	0.963	21.4	-0.27
13	30.9	0.966	21.5	-0.09
6	29.1	0.967	23	-0.76
7	26.6	0.965	23.7	-0.84
29	35.8	0.966	21.8	-1.01
3	29.4	0.965	22.7	-0.63
4	23.1	0.963	24.8	-1.03
16	26.6	0.963	23.5	-0.74
19	25.1	0.961	25.2	-1.56
10	30.9	0.964	23.8	-1.53
8	40.3	0.967	22.5	-2.09
15	33.9	0.962	24	-2.05
11	30.3	0.963	25.5	-2.44
12	43.2	0.967	23.3	-3.00

Table S6 Scoring results for possible tautomeric structures of the ligand AZM in PDB 3HS4 based on the **QM** refinement with AM1.

Structure- 3HS4	SE	RSCC	ZDD	XModeScore
**3**	**17.9**	**0.985**	**17.7**	**2.53**
2	18.7	0.977	27.3	-0.58
1	20.1	0.977	26.8	-1.95
**Resolution 1.6 Å**				
**3**	**18.3**	**0.988**	**5.88**	**2.37**
2	18.9	0.983	10.2	0.13
1	20.4	0.979	12.0	-2.39

Table S7 Scoring results for possible tautomeric structures of the ligand AZM in PDB 3HS4 based on the **conventional** (no QM) Phenix refinement with eLBOW AM1 CIF.

Structure- 3HS4	SE	RSCC	ZDD	XModeScore
**3**	**20.0**	**0.989**	**17.5**	**1.98**
1	35.9	0.988	17.7	-0.75
2	26.2	0.994	18.9	-1.23
**Resolution 1.6 Å**				
2	21.7	0.993	5.72	1.61
**3**	**17.6**	**0.989**	**6.11**	**0.36**
1	28.4	0.989	6.09	-1.97

Table S8 Scoring results for possible tautomeric structures of the ligand AZM in PDB 3HS4 based on the **conventional** (no QM) Phenix refinement with Mogul CIF.

Structure- 3HS4	SE	RSCC	ZDD	XModeScore
**3**	**18.9**	**0.988**	**12.4**	**2.19**
2	19.9	0.991	14	-0.22
1	32.3	0.991	13.8	-1.97
**Resolution 1.6 Å**				
2	18.8	0.992	5.72	0.87
1	30.1	0.992	5.38	-0.32
**3**	**18.1**	**0.988**	**6.33**	**-0.55**

Table S9 Scoring results for possible tautomeric structures of ASP215 in PDB 2JJJ based on the **conventional** (no QM) Phenix refinement with eLBOW CIF for ligand 0QS.

Structure- 2JJJ	SE	RSCC	ZDD	XModeScore
**1**	**47.3**	**0.993**	**2.72**	**0.12**
2	44.9	0.991	4.06	-0.04
3	48.6	0.993	2.23	-0.09

Table S10 Scoring results for possible tautomeric structures of ASP215 in PDB 2JJJ based on the **conventional** (no QM) Phenix refinement with Mogul CIF for ligand 0QS.

Structure- 2JJJ	SE	RSCC	ZDD	XModeScore
**1**	**46.6**	**0.993**	**2.66**	**0.31**
2	44.9	0.991	4.08	-0.12
3	48.3	0.993	2.07	-0.19
